# A new genetic locus for self-compatibility in the outcrossing grass species perennial ryegrass (*Lolium perenne*)

**DOI:** 10.1093/aob/mcaa140

**Published:** 2020-08-28

**Authors:** Lucy M Slatter, Susanne Barth, Chloe Manzanares, Janaki Velmurugan, Iain Place, Daniel Thorogood

**Affiliations:** 1 Institute of Biological, Environmental and Rural Sciences, Gogerddan, Aberystwyth University, Aberystwyth, UK; 2 KWS UK Ltd, Thriplow, Royston, Hertfordshire, UK; 3 Teagasc, Crops Environment and Land Use Programme, Oak Park Research Centre, Carlow, Ireland; 4 ETH Zurich, Department of Environmental Systems Science, Universitätstrasse 2, 8092 Zürich, Switzerland; 5 APC Microbiome Institute, Biosciences Building, University College Cork, Ireland

**Keywords:** Genetic linkage map, genotyping by sequencing, heterosis, inbreeding, Poaceae, quantitative trait locus, reproductive assurance, self-incompatibility, single-nucleotide polymorphism

## Abstract

**Background:**

Self-incompatibility (SI) is a physiological mechanism that many flowering plants employ to prevent self-fertilization and maintain heterozygosity. In the grass family this is known to be controlled by a two locus (*S*-*Z*) system; however, the SI system is intrinsically leaky. Modifier genes of both the *S* and *Z* loci and a further locus, *T*, are known to override SI leading to self-fertilization and self-seed production. This has implications for the ecological and evolutionary success as well as the commercial breeding of grasses. Here we report a study where the genetic control of self-compatibility (SC) was determined from the results of self-pollinating an *F*_2_ population of perennial ryegrass from two independently derived inbred lines produced by single-seed descent.

**Methods:**

*In vitro* self-pollinations of 73 fertile plants were analysed. A genetic association analysis was made with a panel of 1863 single-nucleotide polymorphism (SNP) markers, generated through genotype-by-sequencing methodology. Markers were placed on a recombination map of seven linkage groups (LGs) created using Joinmap v.5. The seed set on self- and open-pollinated inflorescences was determined on 143 plants, including the 73 plants analysed for self-pollination response.

**Key Results:**

Self-pollinations revealed a bimodal distribution of percentage SC with peaks at 50 and 100 %. A single quantitative trait locus (QTL) was identified with peak association for marker 6S14665z17875_11873 that mapped to LG 6. Peak position was associated with maximum marker segregation distortion. The self-compatible plants were equally fecund after self- and open pollination.

**Conclusions:**

This is the first report in the Poaceae family of an SC locus located on LG 6. This new SC QTL discovery, as well as indicating the complex nature of the pollen–stigma recognition process and its evolutionary significance, provides an additional source of SC for breeding perennial ryegrass.

## INTRODUCTION

Self-incompatibility (SI) in perennial ryegrass (*Lolium perenne*), in common with all grasses studied, is controlled gametophytically at the stigmatic surface by two loci, *S* and *Z* (Cornish *et al.*, 1979) located on linkage groups (LGs) 1 and 2, respectively (Thorogood *et al.*, 2002). Although the perennial grass crop species are almost entirely cross-pollinated, many annual grass species, including the major grain crops of the world, are self-pollinating. A non-functional *Z* locus was shown to be responsible for self-compatibility (SC) in the annual species *Lolium temulentum* (Thorogood and Hayward, 1992) and a frameshift mutation of the *L. temulentum* orthologue of the candidate *S* gene, *LpSDUF247*, of *L. perenne* alters the last 24 amino acids of the C-terminus and might explain the failure of the SI system in this species (Manzanares *et al.*, 2016). Self-compatibility in normally self-incompatible grasses is also common (Jenkin, 1931) and the SI system has been shown to be overcome readily by mutations at *S* and *Z* loci (Lundqvist, 1958, 1962, 1968; Thorogood and Hayward, 1991; Hayman and Richter, 1992; Voylokov *et al*., 1993, 1998; Fuong *et al.*, 1993) and at a putative modifier locus on LG 5 (Voylokov *et al.*, 1993; Egorova *et al.*, 2000; Thorogood *et al.*, 2005; Arias-Aguirre *et al*., 2013; Do Canto *et al.*, 2018).

Self-incompatibility in plant populations ensures outcrossing, and high levels of heterozygosity and genetic diversity leading to greater resilience and adaptation to environmental change. For plant breeders, however, the existence of an effective SI system in forage grass crop species is problematic. It means that crop improvement methods are restricted to population improvement strategies. These involve the evaluation of ‘germplasm nurseries’ and the subsequent crossing of selected mother plants forming the basis of a new population that is inherently highly heterozygous and heterogeneous. Following the foundation of the base population, rounds of recurrent selection to fix beneficial traits and to achieve phenotypic uniformity are usually made. In this way favourable genes are accumulated additively. Focus on additive genetic variation may be partially responsible for low improvements in forage grass performance. Genetic gains in annual dry matter yields have been estimated to be ~0.45 % per annum for *L. perenne* and from 0.27 to 0.37 % for *Lolium multiflorum*, amongst the lowest when evaluated against other crops (Laidig *et al.*, 2014; McDonagh *et al.*, 2014). In the early days of maize breeding, cultivars were similarly developed as population synthetics until the discovery of the superior performance of *F*_1_ hybrids (Shull, 1908) that two decades later revolutionized the production of the crop. First implemented in maize, a self-compatible but monoecious crop species, and then adopted in a wide range of crops, *F*_1_ hybrid breeding exploits the enhanced performance (or ‘heterosis’) of a first-generation hybrid resulting from a pair-cross between two inbred lines.

In self-incompatible forage grasses, schemes for the production of ‘50 % hybrids’, where two distinct populations are inter-mated, resulting in up to 50 % of the progeny being of ‘population hybrid’ origin, have been shown to result in heterotic responses (Foster, 1973). By selecting two populations with restricted ranges of contrasting incompatibility alleles it is possible to increase the percentage of population hybrid progeny (England, 1974; Pembleton *et al.*, 2015).

For more efficient exploitation of heterosis, recurrent cycles of self-pollination of self-compatible forage grasses could be made to produce elite inbred parental lines for testing heterotic combinations to maximize yield in *F*_1_ hybrids, as successfully employed in maize (Fernandes *et al.*, 2015). Considerable hurdles still need to be overcome: in producing inbred lines, self-pollination carries with it a fitness penalty because of the high genetic load harboured by outcrossing species that must be purged; physiological mechanisms of pollination control such as cytoplasmic male sterility (Sykes *et al.*, 2017) or chemical emasculation (Hodnett and Rooney, 2018) would need to be developed and implemented.

In perennial ryegrass SC mutations have been selected through obligate selfing of plants and fixed in inbred lines. A classical genetics study in perennial ryegrass characterized pollen tube growth after selfing *F*_2_ populations derived from inbred grandparents (Arias-Aguirre *et al*., 2013). The study concluded that pollen tube behaviour was controlled gametophytically, rendering plants either partially self-compatible (only a proportion of pollen grains were compatible after selfing) or fully self-compatible (100 % of pollen grains were compatible after selfing) and a quantitative trait locus (QTL) on LG 5 was found to be responsible for the SC response. In an earlier study of a similarly constructed but unrelated *F*_2_ family, two genes with major effect were identified co-segregating with the *S*-locus region on LG 1 and at a locus on LG 5 (Thorogood *et al.*, 2005). It is not known if the QTL identified on LG 5 in both studies is controlled by the same locus.

The study reported here was made on a perennial ryegrass *F*_2_ population, unrelated to any population previously used for SC studies. *In vitro* self-pollination and self-seed-setting ability was examined. Genotype-by-sequencing-derived single-nucleotide polymorphic (SNP) markers had been previously genetically mapped (Anhalt *et al.*, 2008; Velmurugan *et al.*, 2016) using Joinmap^®^ 4.1 (Van Ooijen, 2011), which will enable us to associate phenotypic variation for SC with genomic regions.

## MATERIALS AND METHODS

### Recombination mapping

The genotype-by-sequencing SNP marker-mapped ‘*F*_2_ Biomass’ population, described in detail by Anhalt *et al.* (2008), was chosen to investigate the control of SC. The *F*_2_ population was obtained by selfing a single *F*_1_ plant derived from the cross between two unrelated perennial ryegrass inbred lines that had been produced by single-seed descent over nine and ten generations for the maternal and paternal lines, respectively. A total of 1865 segregating SNP markers (Velmurugan *et al.*, 2016) were mapped for 167 *F*_2_ plants using R/qtl (Broman *et al.*, 2003) and JoinMap^®^ 4.1 (Van Ooijen, 2011). A total of 172 (LG 1), 212 (LG 2), 223 (LG 3), 242 (LG 4), 167 (LG 5), 122 (LG 6) and 185 (LG 7) markers were mapped, covering a total map distance of 604 cM. Five hundred and forty-two markers remained unmapped. Individual marker genotypes for the 167 plants are given in Supplementary Data File S1.

Seventy-three individuals were analysed for their *in vitro* self-pollination reaction.

### Pollen compatibility determination

Pollen was collected by placing the inflorescences of plants prior to anthesis within translucent paper bags in a Sanyo Weiss Gallenkamp SGC228 PFX.J Mod (2003) controlled environment (CE) chamber held at 20 °C and barometric pressure 0.6 kPa (74 % relative humidity) with a light intensity of 600 μmol m^−2^ s^−1^. Unpollinated stigmas still attached to their ovary were excised from mature but unopened florets and supported on agar plates (2 % agar agar, 10 % sucrose and 100 ppm boric acid). Self-pollen was dusted onto the stigma surface and the plates were left overnight. Pollen tube behaviour was observed using low-power fluorescence microscopy (Leitz Laborlux K Fluorescence Microscope) on excised pollinated stigmas placed on microscope slides and stained with decolourized aniline blue [0.1 % (w/v) aniline blue in 108 mm K_3_PO_4_]. Slides were scored on a 0–10 scale for pollen compatibility. Only pollen grains producing a small pollen tube tip arrested at or near the stigma surface and large callose occlusions (in accordance with typical stigma surface incompatibility observed in grasses) or pollen grains producing pollen tubes that penetrated and grew into the stigma were scored. Grass pollen is short-lived and viability can be reduced to 5 % in 5 min post-anthesis (Wang *et al.*, 2004), which makes *in vitro* pollination assessments inherently difficult. However, if inactive/unviable pollen grains, showing no signs of pollen tube tip emergence, are excluded from the assessment reasonable estimates of the proportion of compatible to incompatible grains can be made for most *in vitro* pollinations.

### QTL analysis

Analysis of pollen compatibility scores was made with MapQTL^®^ 6 (2009) using multiple regression (Haley and Knott, 1992; Martínez and Curnow, 1992). After selection of markers most significantly associated with QTL identified by multiple regression as cofactors, Multiple QTL Modelling (MQM) mapping (Jansen *et al.*, 1995) was completed to determine the presence of further QTL.

### Seed-set analysis

Seed set was also determined for 143 *F*_2_ plants, including the 73 plants that had been analysed for self-pollination response. Plants were vegetatively propagated to produce three clonal units of each genotype and were grown in an unheated glasshouse in a completely randomized design. Approximately half a dozen non-anthesing inflorescences from each clonal plant unit, still attached to the plant, were tied together with absorbent cotton wool and enclosed in pollen-proof paper ‘crossing bags’, tied and supported to bamboo canes. These were allowed to anthese and the seeds were harvested at full maturity. The remaining inflorescences were left to anthese and mature, unbagged in open-pollinated conditions in the greenhouse. Seed was harvested from the selfed inflorescences and half a dozen or so randomly selected open-pollinated inflorescences from each genotype. Spikelet counts were made for each inflorescence and the mean number of seeds per spikelet was calculated.

## RESULTS

### Phenotypic variation for SC

**Fig. 1.**
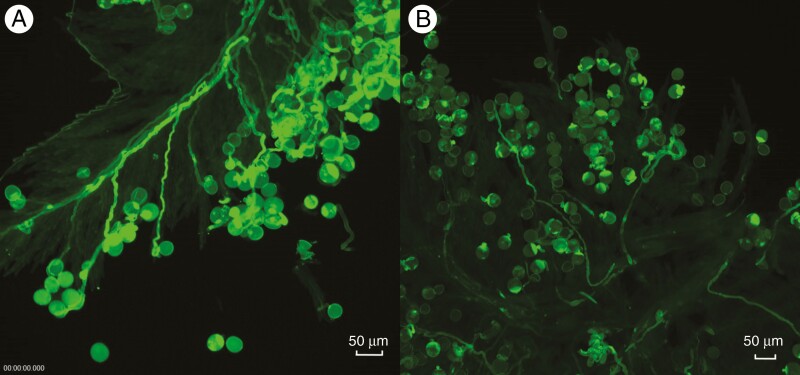
*In vitro* pollination of (A) genotype 212 showing a 100 % self-compatible pollen–stigma reaction and (B) genotype 362 showing a 50 % self-compatible pollen–stigma reaction.

**Fig. 2.**
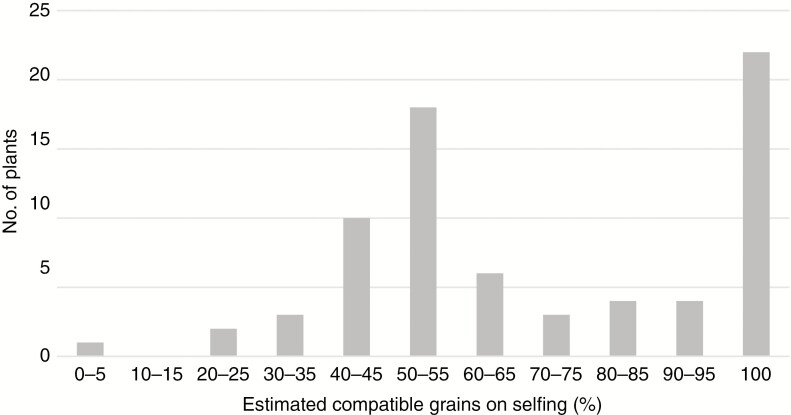
Frequency distribution of estimated percentage SC in the *F*_2_ population.

The mean percentage of compatible pollen grains after self-pollination was estimated for 73 genotypes. Although the distribution was clearly bimodal with 100 and 50 % classes (see Fig. 1 for typical examples) containing 18 and 22 individuals, respectively, a number of pollinations could not be definitively placed in either of these classes and a range of self-pollination fertility scores was obtained (Fig. 2). Individual plant pollen SC scores are listed in Supplementary Data File S2.

### Marker–SC associations

**Fig. 3.**
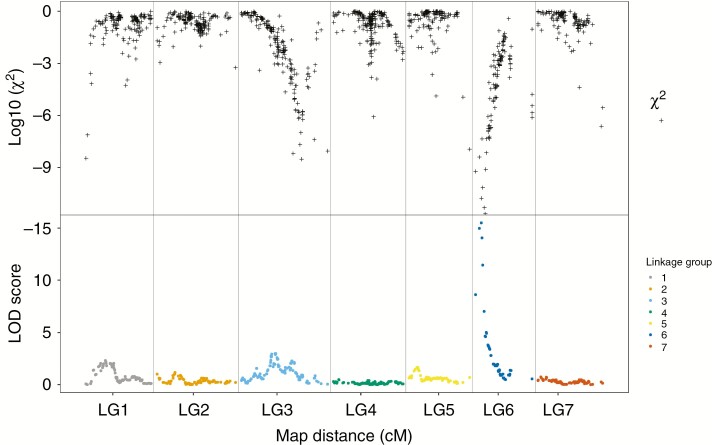
Genome-wide SC QTL analysis. Marker LOD scores are represented as filled colour circles based on analysis of the complete population, and *χ*^2^ test probabilities of marker segregations for agreement with a 1:2:1 ratio are represented as black crosses across seven LGs. The genome-wide permutation test 95 % significance LOD threshold is 3.7.

Initial interval mapping of SC revealed a single major QTL on LG 6 (Fig. 3). The QTL region on LG 6, associated with mean SC scores, with a peak logarithm of the odds (LOD) score of 15.3 at a mapping distance of 10.4 cM, was flanked by two markers (6S142186z594_541 and 6S14665z17875_11873) at 6.4 and 11.2 cM, respectively, with LOD scores of 14.7 and 15.3. This QTL explained 62.0 % of the total variance observed. A proportion of all missing plant marker genotypes were predicted based on genotypes of flanking markers, assuming that double crossovers were unlikely to occur between closely linked markers. Once marker genotypes had been imputed, the two most significantly associated markers only differed in one plant, suggesting that a single crossover event between the two markers might have occurred. The mean pollen compatibility scores for aa, ab and bb genotypes for marker 6S14665z17875_11873 were 47.0, 50.6 and 93.6 %, respectively.

The segregation ratio of the closest linked markers deviated significantly from the expected 1:2:1 ratio, though the ratio of homozygous to heterozygous individuals agreed with the expected 1:1 ratio (Table 1). We performed *χ*^2^ tests for 1:2:1 ratios for all markers and log_10_ probability scores were plotted against recombination distance (Fig. 3). The marker SNP alleles G (6S142186z594_541) and A (6S14665z17875_11873) were transmitted at a greater rate than the alternative alleles, A and G, respectively, and were derived from the maternal parent. Many markers throughout the genome deviated significantly from the expected ratio, but only markers on LGs 3, 4 and 6 formed distinct genomic regions, with marker distortion that produced maximum *χ*^2^ probabilities at 100.5, 66.5 and 14.5 cM, respectively. The region with maximum distortion on LG 6 coincided with the peak position of the SC locus, dissolving incrementally with increasing genetic distance from the peak position.

**Table 1.** SNP genotypes of parents and *F*_1_ and *F*_2_ segregation ratios for the two SNP markers most closely linked to the SC locus on LG 6

	SNP marker					
	6S142186z594_541			6S14665z17875_11873		
Parents	GG	X	AA	AA	X	GG
*F*_1_		AG			AG	
*F*_2_ segregants	AA	AG	GG	AA	AG	GG
	7	68	66	60	51	4
*P* for 1:2:1		4.08 × 10^−9^			1.74 × 10^−11^	
*P* for 1:1 (homozygous:heterozygous)		0.64			0.67	

Full details of *F*_2_ genotyping can be found at https://ics.hutton.ac.uk/jbrowse/lolium, filtered for ‘SNPs from GBS study’.

A, adenine; G, guanine.

**Fig. 4.**
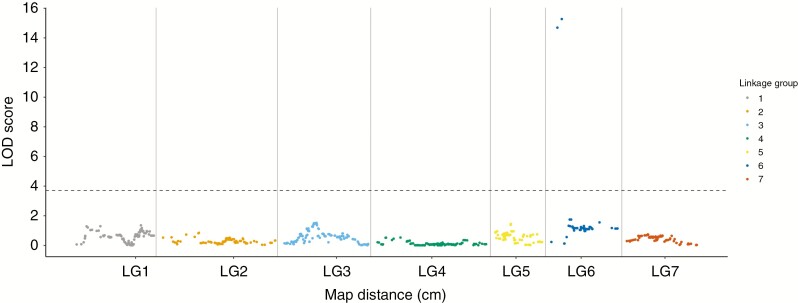
Genome-wide SC MQM analysis made after selecting marker 6S14665z17875_11873 as a cofactor. The genome wide permutation test 95 % significance LOD threshold is 3.8.

As there were phenotypic differences in the proportion of pollen grains that were compatible in partially self-compatible pollinations and the LG 6 QTL identified explained a large proportion of the total variance, a further MQM analysis, to identify relatively small additive and dominant gene actions, was made after selecting marker 6S14665z17875_11873 as a cofactor. A QTL was still observed on LG 6 with the same magnitude as in the original QTL analysis but with a narrower peak focused around the marker 6S14665z17875_11873 (LOD score 15.3). No further QTL were revealed across the rest of the genome (Fig. 4).

### Seed-setting ability

**Fig. 5.**
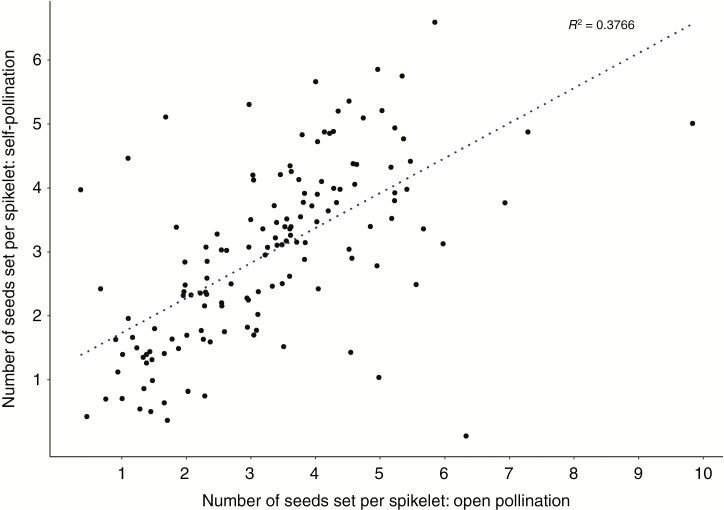
Correlation between mean numbers of seeds set per spikelet for plants self-pollinated and open-pollinated (*R* = 0.61).

The mean number of seeds per spikelet in selfed (*x̄* = 3.00, *n* = 142) and open-pollinated (*x̄* = 3.28, *n* = 141) plants was not statistically different (*P* = 0.09, Student’s *t*-test) and there was a positive correlation (*R* = 0.61; *n* = 140) between seed set on genotypes selfed and open-pollinated (Fig. 5). Furthermore, there was no correlation between SC score and seed set on selfing (*R* = −0.16).

## DISCUSSION

Self-incompatibility is known to break down in many flowering plant species either due to loss-of-function mutations at *S* loci or other multiple loci involved in the SI response (Igic *et al.*, 2008). Extensive literature points towards SC being a complex trait that includes genetic control by many genes that modify the SI response. The transition from outcrossing to selfing is one of the most prevalent evolutionary trends in flowering plants (Stebbins, 1974). The transition is irreversible and the process provides reproductive assurance for colonizing and ruderal species in accordance with Baker’s law (Cheptou, 2012). Although there are many examples of self-compatible *Lolium* species, for perennial ryegrass the ability to outcross is largely retained, with most populations studied, although mostly consisting of self-incompatible individuals, containing a proportion of plants able to set seed on selfing (see Jenkin, 1931 for early examples). It is clear that SC commonly occurs in natural ryegrass populations: the process of inbreeding by obligate selfing, as used in the production of the two parents for the *F*_2_ population used in the current study and the studies of Thorogood *et al.* (2005) and Do Canto *et al.* (2018), provides the selection pressure needed to fix the naturally induced mutations responsible for SC at loci other than the SI loci, *S* and *Z*. Although there appear to be no recorded examples of SC mutations at the SI (*S* and *Z*) loci themselves in *Lolium*, mutations at both *S* and Z were reported in the self-incompatible grass species *Secale cereale* (Voylokov *et al.*, 1993). Overall, it can be concluded that a range of SC genes, either associated directly with the SI loci or as unlinked modifier loci, exist within largely self-incompatible species and enable populations to retain genetic diversity and at the same time have the ability to colonize new habitats where mate choice might be restricted, potentially extending the natural range of the species. This flexible reproductive strategy may well be partly responsible for the success of *L. perenne*, which has a wide geographical distribution (Beddows, 1967) that, although accelerated by agricultural use, has occurred through natural processes predating primitive agriculture (Harper *et al.*, 2019; Blanco-Pastor *et al.*, 2019).

This study reports the action of a newly discovered *S-Z* SI modifier locus responsible for rendering a normally outcrossing grass species, perennial ryegrass, self-fertile. The locus is located on LG 6 and the phenotypes and segregation patterns observed are similar to those expressed in two unrelated *F*_2_ populations reported to be segregating for SC loci on LG 5 (Thorogood *et al.*, 2005; Do Canto *et al.*, 2018).

The heterozygous phenotypes arise, through dierential transmission of the pollen gametophytes of the *F*_1_ plant, as partially self-compatible plants: the pollen grains not possessing the SC gene are arrested immediately as the germinating pollen tubes make contact with the stigma surface, presumably controlled by matching *S* and *Z* alleles expressed in the pollen grain and the receiving stigma. This results in the non-transmission of the wild-type allele and subsequent distorted segregation of linked markers in self-progeny, which is reduced over genetic distance from the SC gene. The deficient alleles of the linked markers with low transmission were derived from the paternal parent (Table 1), demonstrating that the SC gene derives from the maternal parent. The region delimited by the marker with the highest LOD score (6S14665z17875_40584) and its two flanking markers (6S142186z594_541 and 6S4103z49456_11373) covers a recombination distance of 7.4 cM and a physical region of ~13.77 Mbp that contains 736 gene predictions (D. Thorogood, unpubl. data).

In the current study, even when applying a cofactor to the LG 6 QTL marker 6S14665z17875_40584 and implementing an MQM mapping analysis, no further significant QTL were identified. The very nature of inbreeding by obligate selfing would indicate that both inbred lines used to produce the *F*_2_ population would have been self-compatible. In our 2005 study (Thorogood *et al.*, 2005) of an unrelated *F*_2_ population, two segregating SC QTL were identified: the so-called *T*-locus on LG 5 and an *S*-locus-linked QTL on LG 1 derived from the maternal and paternal lines, respectively. The fact that we were unable to identify an SC factor deriving from the paternal inbred line in the current study suggests this line was maintained through pseudo-self-compatibility controlled either by polygenic modifiers each with minor effect that are undetectable in small populations (Levin, 1996) or by an environmental factor such as high temperature (Wilkins and Thorogood, 1992).

Three genomic regions were identified on LGs 3, 4 and 6 where patterns of distortion consistent with a genetic effect were observed. As opposed to marker-scoring interpretation errors, there are many genuine biological reasons for segregation distortion of markers, such as preferential fertility, fitness and fecundity (Xu *et al.*, 1997). In these cases maximum marker distortion is displayed by those markers closest to the position of the functional segregation distortion locus, with an incremental reduction in its significance moving away from this point. The coincidence of segregation distortion and SC phenotype on LG 6 leads to a powerful approach to map-based gene discovery based on the degree of marker segregation distortion caused by the differential response of the pollen gametophytes in a large single bulk population.

The favoured allele of the marker, 6S14665z17875_40584, most closely associated with SC on LG 6, was derived through the maternal line. This marker sequence contains a 241-bp sequence that aligns with the *Brachypodium distachyon* gene BRADI3g54387 with 92.1 % similarity. This gene encodes a diacylglycerol glucosyltransferase protein involved in glycolipid biosynthesis (GO: 0016758; GO: 0009247). Although glycolipids function in cell membrane stability and cell recognition, an important component of the SI reaction, it is unlikely that this gene is a genuine candidate as four of 115 plants were homozygous GG and must have been formed as a result of recombination between the marker and the SC locus in the pollen gametes. A physical *L. perenne* pseudomolecule assembly is currently being developed and we estimate that the QTL region contains over 700 gene predictions. Our ongoing approach to narrowing this region to a small number of candidate genes will be to carry out a bulk segregant analysis (BSA) of the two SC phenotypes in this population using genotype-by-sequence-based SNP markers and to associate allele frequencies with the proximity of markers to the functional gene. Subsequent gene expression studies and modelling of candidate gene function will help to elucidate genetic, molecular and cellular processes involved in controlling the grass SI system. At the same time, an approach whereby a single bulk population is screened for high-read-depth marker allele frequencies could be used to identify markers with the most highly distorted frequencies. The advantage of a single bulk method would be that it negates the need for phenotyping in conventional BSA, which is prone to phenotype misclassification errors influencing map accuracy.

There is a significant amount of research on the effect of marker segregation distortion on mapping and, recently, investigations in soybean mapping (Zuo *et al*., 2019) demonstrated that the inclusion of distorted markers improved the consistency of marker map order but also increased genome coverage. Moreover, there are a number of examples of distorted segregation associated with a range of different SI systems, for example sporophytic SI in *Arabidopsis lyrata* (Bechsgaard *et al.*, 2004) and gametophytic SI in *Schlumbergera* (Cactaceae) (O’Leary and Boyle, 1998), *Petunia hybrida* (Harbord *et al.*, 2000) and *Prunus armeniaca* (Zuriaga *et al.*, 2013).

For any practical application of SC, pollen tube growth must ultimately result in fertilization and seed development. Limiting pollen availability to just self-pollen resulted in the same amounts of seed being set as in open-pollinated inflorescences even when plants were only partially self-compatible. This demonstrates that selfing had no deleterious effect on seed-setting ability and self-pollen had no observable effect on the plants’ fecundity. Even in partially self-compatible pollinations there was no indication that a lower proportion of compatible pollen limited seed setting ability or that self-incompatible pollen on the stigma surface impeded self-compatible pollen growth and successful self-fertilization.

The discovery also opens up opportunities to develop inbred lines and doubled haploid populations useful for studying inbreeding depression and heterosis. For SC to be of practical use in developing first-generation hybrid forage and turfgrass varieties, the challenges of purging deleterious alleles and selecting elite inbred lines and developing effective pollination control strategies for *F*_1_ hybrid production remain to be solved.

## SUPPLEMENTARY DATA

Supplementary data are available online at https://academic.oup.com/aob and consist of the following. File S1: individual marker genotypes for the 73 *F*_2_ plants used for QTL analysis. File S2: individual plant pollen SC scores.

mcaa140_suppl_Supplementary_Material_S1

mcaa140_suppl_Supplementary_Material_S2

## FUNDING

This work was supported by the UK Biotechnology and Biological Sciences Research Council through Institute Strategic Programme and Core Strategic Programme grants (BB/J004405/1, BB/CSP1730/1 and BB/G012342/1).
